# Impact of pre-existing comorbidities on outcomes of patients undergoing surgical aortic valve replacement – rationale and design of the international IMPACT registry

**DOI:** 10.1186/s13019-021-01434-w

**Published:** 2021-03-25

**Authors:** Farhad Bakhtiary, Ali El-Sayed Ahmad, Rüdiger Autschbach, Peter Benedikt, Nikolaos Bonaros, Michael Borger, Oliver Dewald, Richard Feyrer, Hans-Joachim Geißler, Jürg Grünenfelder, Ka Yan Lam, Rainer Leyh, Andreas Liebold, Markus Czesla, Arash Mehdiani, Francesco Pollari, Saad Salamate, Justus Strauch, Andreas Vötsch, Alberto Weber, Daniel Wendt, Beate Botta, Peter Bramlage, Andreas Zierer

**Affiliations:** 1 Division of Cardiac Surgery, Heart Center Siegburg-Wuppertal, University Witten-Herdecke, Ringstr. 49, 53721 Siegburg, Germany; 2 University Hospital RWTH Aachen, Aachen, Germany; 3 Kepler University Hospital Linz, Linz; and Hospital Wels-Grieskirchen, Wels, Austria; 4 Medical University of Innsbruck, Innsbruck, Austria; 5 University Hospital Leipzig, Leipzig, Germany; 6 University Hospital Oldenburg, Oldenburg, Germany; 7 Bundeswehrzentralkrankenhaus, Koblenz, Germany; 8 Heart Clinic Hirslanden, Zurich, Switzerland; 9 Catharina Hospital Eindhoven, Eindhoven, The Netherlands; 10 University Hospital Wuerzburg, Wuerzburg, Germany; 11 University Hospital Ulm, Ulm, Germany; 12 Hospital Passau, Passau, Germany; 13 University Hospital Duesseldorf, Duesseldorf, Germany; 14 Cardiac Surgery, Klinikum Nürnberg – Paracelsus Medical University, Nuremberg, Germany; 15 University Hospital Bergmannsheil, Bochum, Germany; 16 Department of Cardiovascular and Endovascular Surgery, Paracelsus Medical University, Salzburg, Austria; 17 Heart Center Hirslanden, Zurich, Switzerland; 18 Essen University Hospital, Essen, Germany; 19 Institute for Pharmacology and Preventive Medicine, Cloppenburg, Germany

**Keywords:** Aortic valve disease, Surgical aortic valve replacement, SAVR, Comorbidities, Real-world setting

## Abstract

**Background:**

Degenerative aortic valve disease accounts for 10–20% of all cardiac surgical procedures. The impact of pre-existing comorbidities on the outcome of patients undergoing surgical aortic valve replacement (SAVR) needs further research.

**Methods:**

The IMPACT registry is a non-interventional, prospective, open-label, multicenter, international registry with a follow-up of 5 years to assess the impact of pre-existing comorbidities of patients undergoing SAVR with the INSPIRIS RESILIA aortic valve on outcomes. IMPACT will be conducted across 25 sites in Austria, Germany, The Netherlands and Switzerland and intends to enroll approximately 500 patients. Patients will be included if they are at least 18 years of age and are scheduled to undergo SAVR with the INSPIRIS RESILIA Aortic Valve with or without concomitant ascending aortic root replacement and/or coronary bypass surgery. The primary objective is to determine all-cause mortality at 1, 3, and 5 years post SAVR. Secondary objectives include cardiac-related and valve-related mortality and structural valve deterioration including hemodynamics and durability, valve performance and further clinical outcomes in the overall study population and in specific patient subgroups characterized by the presence of chronic kidney disease, hypertension, metabolic syndrome and/or chronic inflammation.

**Discussion:**

IMPACT is a prospective, multicenter European registry, which will provide much-needed data on the impact of pre-existing comorbidities on patient outcomes and prosthetic valve performance, and in particular the performance of the INSPIRIS RESILIA, in a real-world setting. The findings of this study may help to support and expand appropriate patient selection for treatment with bioprostheses.

**Trial registration:**

ClinicalTrials.gov identifier: NCT04053088.

## Background

Degenerative aortic valve disease (DAVD) is a condition that leads to deterioration of the heart valves and is an emerging health problem with broad consequences for the patient and the healthcare system alike [1]. Treatment for DAVD is either transcatheter aortic valve implantation (TAVI) or surgical aortic valve replacement (SAVR) with either mechanical or bioprosthetic valves [2].

Within this context, it is known that risk factors, like diabetes, low-density lipoprotein (LDL) cholesterol, hypertension and smoking, increase the likelihood of developing DAVD [3]. It is also clear that some treatments, like SAVR or TAVI, confer risk for direct postoperative complications, such as the development of acute kidney injury, which may affect patient survival [4, 5]. But the impact of pre-existing comorbidities on the outcome of patients undergoing SAVR is less clear. Not only are comorbidities associated with surgical complications, but they also impact on the quality of the patient’s recovery and their long-term functional recovery after surgery [6]. A study to determine the impact of comorbid illness on mortality outcomes after cardiac surgery has shown that diabetes, vascular disease, chronic obstructive pulmonary disease (COPD), peptic ulcer disease and renal failure significantly increase the risk of death after coronary artery bypass grafting (CABG) [7]. Another recent study has shown that comorbidity, as measured by the Charleston comorbidity index (CCI), is an independent risk factor for both 1- and 3-year mortality in patients undergoing SAVR [8]. While more work is needed to establish which comorbidities will impact on the outcome of SAVR, there are certain known comorbidities that are very likely to have a negative impact on the outcome of SAVR – these include renal dysfunction, pulmonary hypertension and diabetes [9–11]. Thourani et al conducted a large-scale study to determine the impact of renal dysfunction – ranging from mild renal dysfunction (glomerular filtration rate [GFR] 60–90 mL/min) to severe renal dysfunction (GFR 15–30 mL/min) – on SAVR and concluded that the presence of pre-existing renal dysfunction is associated with poor prognosis. Furthermore, the more severe the renal dysfunction, the worse the outcome post-SAVR [10]. The impact of diabetes, however, warrants further investigation. Diabetes is typically associated with worse outcomes after surgery [11, 12]. Surprisingly, however, a study of patients with and without type 2 diabetes mellitus (T2DM) undergoing either TAVI or SAVR suggested that the prognosis of patients with T2DM was not different to non-diabetic patients [13].

While mechanical valves have been the mainstay of SAVR, the use of bioprostheses for the surgical treatment of valve diseases has increased considerably over the last decade [14]. They were introduced to improve the hemodynamic performance of the valves and to reduce the risk of structural valve deterioration (SVD) [15]. This was accompanied by improvements of tissue preservation technologies, which resulted in a reduction of intracellular calcium infiltration, stiffening and weakening of the tissues. There is, however, still a lack of data on the impact of patients’ predisposing risk factors such as chronic kidney disease (CKD) [16] diabetes and the metabolic syndrome (MS) [17] and age. However, literature on this topic is scattered and data are scarce.

Bioprosthetic valve durability cannot, therefore, be generalized as it is influenced by the type and design of the implanted prosthetic valve, and the particular physiology of the patient. To better understand the impact of pre-existing comorbidities on the outcome of SAVR using bioprosthetic valves, we developed a multicenter registry to gather long-term follow-up data on patients implanted with the INSPIRIS RESILIA aortic valve concerning SVD and valve performance, with a particular focus on patients with pre-existing chronic kidney disease, diabetes, hypertension, metabolic syndrome or chronic inflammation. Information from this study will enable clinicians to make informed decisions regarding the use of SAVR in comorbid patients.

## Methods/design

The IMPACT registry is a non-interventional, prospective, open-label, multicenter, international registry with a follow-up of 5 years to assess the impact of pre-existing comorbidities on outcomes of patients undergoing surgical aortic valve replacement (SAVR) with the INSPIRIS RESILIA aortic valve (Edwards Lifesciences). An estimated 500 patients will be enrolled across 25 sites in Germany, Austria, the Netherlands and Switzerland, with a mean of 20 patients per site enrolled over a 12-month period. Site selection is based on recommendations of the steering committee and on the site’s prior SAVR experience. The registry will be conducted in accordance with the European Medical Device Regulation (Regulation (EU) 2017/745 of 5 April 2017) and ISO 14155:2011. Patients will need to provide written, informed consent prior to enrolment.

### The valve

The INSPIRIS RESILIA aortic valve, is a stented trileaflet valve comprised of RESILIA bovine pericardial tissue that is mounted on a flexible frame. The valve is stored under dry packaging conditions and consequently does not require rinsing prior to implantation. RESILIA tissue is the result of a technology that incorporates a stable-capping anticalcification process permanently blocking residual aldehyde groups that are known to bind with calcium. It also includes tissue preservation with glycerol, which replaces the traditional storage in liquid-based solutions such as glutaraldehyde. As such leaflet calcification and hemodynamic performance is improved [18]. The frame is designed to be compliant at the orifice as well as at the commissures. The wireform is made of a corrosion-resistant cobalt-chromium alloy to improve spring efficiency and fatigue-resistance and is covered with a polyester fabric. Finally, VFit technology, which includes fluoroscopically visible size markers and an expansion zone, eases potential future valve-in-valve (ViV) procedures.

### Patients

Patients over the age of 18 years undergoing SAVR and receiving the INSPIRIS RESILIA Aortic Valve will be enrolled on a consecutive basis. In addition to the applicable criteria of the device Instructions for Use (IFU), the registry inclusion criteria stipulate that patients have an aortic valve vitium and require aortic valve replacement, and are scheduled to undergo SAVR using the INSPIRIS RESILIA aortic valve with or without concomitant ascending aortic root replacement and/or coronary bypass surgery. Patients are scheduled to attend yearly follow-up visits on site for up to five years.

Patients will be excluded if they have active endocarditis or myocarditis, or had endocarditis or myocarditis within three months prior to the scheduled SAVR, if valve implantation is not possible in accordance with the device IFU, the patient has a life expectancy of less than 12 months for any reason, or the patient is pregnant at the time of the surgery.

### Objectives

The primary objective of the study is to determine all-cause mortality at 1, 3, and 5 years after SAVR (Table 1). The secondary objectives are designed to assess the study population mortality, hemodynamics and durability of the prosthetic valve, and clinical outcomes. Study population mortality is defined as all-cause, cardiac- and valve-related mortality in the overall study population and in the following patient subgroups at all time points: patients with chronic kidney disease, diabetes, hypertension, metabolic syndrome and chronic inflammation. Assessment of hemodynamics and durability will include the hemodynamic performance of the INSPIRIS RESILIA aortic valve in the overall study population and stratified according to the different patient subgroups including patient prosthesis mismatch (PPM) including maximum (Pmax) and mean (Pmean) pressure gradient across the valve, velocity time integral (VTI), prosthesis opening area (EOA), left ventricular ejection fraction (LVEF) and paravalvular leak (PVL); severe hemodynamic SVD following Salaun [19]; and potential valve-in-valve (ViV) procedures and clinical outcome including follow-up. Clinical outcomes will be assessed in the overall study population and, in addition, will be stratified across the following patient subgroups: New York Heart Association (NYHA) functional class compared to baseline, freedom from valve-related rehospitalization, new pacemaker implant and freedom from valve-related complications. Exploratory safety endpoints are presented in Table 1.

**Table 1:** Registry objectives

Primary objectives	To determine all-cause mortality 1, 3, and 5 years after surgery
**Secondary objectives**	**Mortality**
	To determine all-cause, cardiac- and valve-related mortality in the overall study population and in different patient subgroups at all timepoints:
	• Chronic kidney disease
	• Diabetes
	• Hypertension
	• Metabolic syndrome
	• Chronic inflammation
	**Hemodynamic performance and durability**
	• Hemodynamic performance of the INSPIRIS RESILIA Aortic Valve™ in the overall study population and stratified according to the different patient subgroups including patient prosthesis mismatch (PPM) including maximum (Pmax) and mean (Pmean) pressure gradient across the valve, Velocity Time Integral (VTI), Prosthesis Opening Area (EOA), Left Ventricular ejection fraction (LVEF), Paravalvular Leak
	• Severe hemodynamic structural valve deterioration (SVD) following Salaun [19]
	• Description of potential ViV procedures* and clinical outcome including follow-up
	**Clinical outcomes** (overall study population and stratified according to the different patient subgroups)
	• NYHA functional class compared to baseline
	• Freedom from valve-related rehospitalization
	• New pacemaker implantation
	• Freedom from valve-related complications
**Exploratory endpoints**	**Safety (defined according to VARC-2)**
	• SVD
	• Non-structural valve deterioration
	• Thromboembolic events (e.g., stroke)
	• Valve thrombosis
	• All bleeding/hemorrhage
	• Major bleeding/hemorrhage
	• All paravalvular leakage
	• Endocarditis
	• All-cause mortality
	• Cardiac-related mortality
	• Valve-related mortality
	• Re-intervention
	• Conduction disturbances

*Legend:* NYHA, New York Heart Association; SVD, structural valve degeneration; VARC, Valve Academic Research Consortium; ViV, valve-in-valve. *Valve-in-valve procedures include both transcatheter aortic valve implantations (TAVI) in surgical aortic valve replacements (SAVR) and SAVR in SAVR replacements

### Data collection

The clinical outcome data collected will be based on the site’s standards of care for SAVR. The collected data will include medical history, physical assessments, safety parameters, electrocardiogram (ECG), laboratory results and transthoracic/transesophageal echocardiography (Table 2). Data will be captured by an electronic case report form (eCRF; Software for Trials Europe GmbH, Berlin, Germany) and data will be checked automatically for plausibility and completeness.

**Table 2:** Data collection schedule

	Screening/baseline	Surgery	Discharge	Year 1	Year 3	Year 5
Medical history^1^	X					
Physical examination^2^	X		X	X	X	X
ECG (12-lead)	X		X	X	X	X
Echocardiogram (TTE)	X		X	X	X	X
Core Lab echo				X		X
NYHA class/CCS angina class	X			X	X	X
Medications	X		X	X	X	X
Procedural information		X				
Aetiology		X				
SAE reporting		X	X	X	X	X
Discharge data			X			
Rehospitalization data^3^				X	X	X
Return to work				X	X	X

^1^Includes cardiovascular and non-cardiovascular conditions, prior cardiac interventions and surgeries

^2^Physical examination, includes height, weight and vital signs (blood pressure and heart rate)

^3^Includes re-interventions, potential ViV procedures

*Legend:* CCS, Canadian Cardiovascular Society; ECG, electrocardiogram; NYHA, New York Heart Association; SAE, serious adverse event(s); TTE, transthoracic echocardiogram

### Echocardiography core lab

Digital imaging and communication in medicine (DICOM) files of the echocardiograms generated at years 1 and 5 will be collected for analysis by the Echo Core Laboratory to ensure unbiased and consistent analysis of the diagnostic data and, by using serial echocardiographic studies conducted on the same patient, for evaluating changes in patient status over the course of the registry.

### Monitoring

Physicians/surgeons and study personnel are required to make themselves familiar with the registry protocol, eCRF, requirements and procedures. Approximately 20% of sites will be selected at random and monitored. In these centers, source data verification will be performed for all patients.

### Statistical analysis

The study sample size was determined based on feasibility considerations. Furthermore, 95% confidence intervals (CIs) for the primary endpoint at different event rates were determined and taken as a basis for the final sample size. It was estimated, from the COMMENCE Trial dataset where all-cause mortality was determined to be 1.2% at year 1 and 2.0% at year 2 [20], that 500 patients will arrive at a 95% confidence interval (CI) of ±0.87 for a 1% observed event rate and ± 1.50 for a 3% observed event rate.

Statistical analyses will be performed for the total study population and for the subgroups defined by baseline comorbidities if applicable. Continuous variables will be presented as mean ± standard deviation (SD) or as median with interquartile range (IQR), and categorical variables (e.g., gender) will be reported as frequencies and percentages. The Kolmogorov-Smirnov test will be used to test for normal distribution. Accordingly, comparisons will be performed using Student’s t-test or Mann-Whitney U test for continuous variables. The Chi-Square or Fisher exact test will be used for categorical variables. Linearized rates and actuarial probability statistics may be used where appropriate for adverse event reporting. Kaplan-Meier analyses will be performed for survival and safety outcomes. All statistical analyses will be performed using IBM SPSS Statistics version 24 (IBM, Armonk, New York) or R Core Team (https://www.R-project.org/), with a *p*-value of < 0.05 considered statistically significant.

## Discussion

The IMPACT registry has been designed to provide prospectively collected data that can be used to elucidate the benefits and risks of the surgical implantation of INSPIRIS RESILIA in patients with pre-existing comorbidities, as well as the long-term hemodynamic performance and durability of the valve in these patients. The findings of this registry will provide much-needed information on the impact of bioprosthetic valves in patients with concomitant morbidities.

### Comorbidities

The age of the global population is steadily increasing [21], which also means that the risk of DAVD is rising [22]. Increasing age also increases the likelihood of patients having additional comorbidities and it is these pre-existing comorbidities in patients undergoing surgery that affect the risk of serious complications [23–25]. It is known that comorbidities are an independent risk factor for both 1- and 3-year mortality in patients undergoing SAVR [8], but there is limited data on which specific comorbidities impact this risk. There is a trend for patients with comorbidities to undergo TAVI in preference to SAVR to mitigate the risk of treatment [26]. As such, there is a clear need to determine whether pre-existing comorbidities impact on patient outcomes or prosthetic valve performance after SAVR in a real-world setting and, if so, which comorbidities are of paramount concern. The aim of this registry is to provide this much-needed information as it will allow clinicians to make research-backed, informed decisions about which patients may benefit from treatment with SAVR.

### Determinants and surrogates of valve failure

The overall aim of bioprosthetic valves is to provide uncompromised hemodynamic function, which is durable for many years, with no evidence of structural degeneration necessitating valve replacement, ViV interventions or death [27, 28]. For this current registry, the definition of SVD provided by Salaun [19] has been adopted as a surrogate for valve degeneration because it incorporates terminology proposed by both Dvir [29] and Capodanno [30] and was compatible with the definition used by Pibarot et al. [31]. We will, however, aim to capture the components of the other definitions such that these can be also be compared.

### IMPACT in perspective

Two ongoing registries – RESILIENCE and INDURE [31, 32] – will assess the long-term performance and structural integrity of bioprosthetic valves using the RESILIA tissue in younger patients. A comparison of these studies with the IMPACT registry is shown in Table 3. IMPACT, INDURE and RESILIENCE are all prospective studies and include patients receiving either the INSPIRIS RESILIA valve (IMPACT and INDURE) or any valve bearing RESILIA tissue (RESILIENCE). Both the IMPACT and INDURE registries will follow patients from the time of surgery for up to 5 years, while RESILIENCE pursues retrospective inclusion of patients with the first visit being 5-years after surgical intervention and a prospective follow-up (up to year 11 post implant). The patient populations for the studies are different, with IMPACT being the only study to focus on patients with pre-existing comorbidities, including chronic kidney disease, diabetes, hypertension, metabolic syndrome and inflammation. The outcomes of the three studies are varied. IMPACT assesses all-cause mortality at years 1, 3 and 5 post-surgery; INDURE measures time-related valve safety at 1-year, SVD defined according to Salaun [19] using a CoreLab and clinical outcomes; and RESILIENCE focuses on the multi-slice computed tomography (MSCT) and echo-based (both CoreLab) prediction of re-intervention or valve-related death. Projected completion dates are 2025 (INDURE), 2026 (IMPACT) and 2027 (RESILIENCE), respectively. The data from the IMPACT registry will complement the data derived from both INDURE and RESILIENCE.

**Table 3:** IMPACT vs. INDURE vs. RESILIENCE

	IMPACT (NCT04053088)	INDURE (NCT03666741)	RESILIENCE (NCT03680040)
Valve used	INSPIRIS valves	INSPIRIS valves	RESILIA tissue valves
Design	Prospective	Prospective	Retrospective inclusion, prospective follow-up
Study Start date	12 December 2019	26 April 2019	5 November 2018
Baseline	Implantation	Implantation	5 years
Follow-up	5 years – projected completion 2026	5 years – projected completion 2025	6 years (from year 5 to year 11) – projected completion 2027
Subjects/centers	500 subjects, ~ 25 centers (Austria, Germany, The Netherlands and Switzerland), patients ≥18 years at the time of their SAVR	400 subjects, 20–25 centers (EU and Canada), patients ≤60 years at the time of their SAVR	220 subjects, up to 15 centers (US and EU), patients ≤65 years at the time of their SAVR
Objective	Assess clinical outcomes in patients with pre-existing comorbidities	Assess clinical outcomes	Time to valve failure due to valve degeneration requiring re-intervention & early potential predictors of valve durability
Primary endpoints	All-cause mortality at 1, 3 and 5 years after SAVR	Time-related valve safety at 1 year (VARC-2)	Time to BVF due to SVD, defined as requiring re-intervention (redo surgery or ViV), or confirmed valve related death, according to Akin criteria [27]
		Rate of severe SVD (stage 3 following Salaun [19]) at 5 years (Echo CoreLab)	
Secondary endpoints	Mortality (all-cause, cardiac-related and valve-related) in overall population and patient subgroups Hemodynamics and durability Clinical outcomes (NYHA; freedom from rehospitalization, new pacemaker implant and valve-related complications)	Hemodynamics and durability (Echo CoreLab) Clinical outcomes (NYHA and freedom from rehospitalization) Quality-of-life (KCCQ & SF-12)	Early possible predictors of valve failure including leaflet calcification and morphological/hemodynamic valve degeneration: - Valve leaflet calcification via CoreLab evaluated MSCT (no contrast) - Hemodynamic performance (Echo CoreLab)

*Legend:* BVF, bioprosthetic valve failure; EU, European Union; KCCQ, Kansas City Cardiomyopathy Questionnaire; MSCT, multi-slice computed tomography; NYHA, New York Heart Association; SF-12, Short Form-12; SVD, structural valve degeneration; US, United States; VARC, Valve Academic Research Consortium; ViV, valve-in-valve

### Appreciation of the study design

IMPACT is a prospective, multicenter European registry, involving centers from Austria, Germany, The Netherlands and Switzerland. The multinational nature of this registry will increase the applicability of its findings to other European and international countries. Due to financial limitations, there is no control group with other types of bioprosthetic valves, making a comparison of valve types or valve generations impossible. However, long-term follow-up data with an assessment of patient outcomes and valve deterioration are scarce; an investigation into the impact of comorbid conditions in patients undergoing valve replacement is valuable and will enable the responsible use of this valve type in clinical practice. Furthermore, comparison between bioprosthetic valves and mechanical valves is beyond the scope of this registry. The multicenter design necessitated the establishment of a uniform assessment of SVD to be used over the 5-year follow-up. While five years is a considerable time-span for the study to judge mortality, it is also possible that early signs of valve deterioration may occur during this timeframe. This study has been designed in such a way that, with appropriate financial support, it can be extended beyond the proposed 5-year follow-up should it be possible to achieve reasonable data completeness. Data completeness, however, can be challenging in these patients when the survival for patients aged ≥75 years post SAVR with a bioprosthesis is 6–7 years [33]. It was not possible to establish an MCST CoreLab, as per the RESILIENCE trial, as this would have violated the non-interventional nature of the IMPACT registry and MSCT is not the standard-of-care for all sites used in this registry. Where MSCT is used in routine practice, this data is documented. Finally, the same INSPIRIS RESILIA valve will be used in all patients in the INDURE registry which will abolish any bias introduced by the use of different bioprosthetic valves.

## Conclusions

IMPACT is a prospective, multicenter European registry designed to provide much-needed data on the impact of pre-existing comorbidities on patient outcomes post SAVR and prosthetic valve performance, and in particular the performance of the INSPIRIS RESILIA, in a real-world setting. The findings of this study may help to support and expand appropriate patient selection for treatment with bioprostheses.

## Data Availability

Not applicable.

## Abbreviations

DAVD: degenerative aortic valve disease
DICOM: digital imaging and communication in medicine
ECG: electrocardiogram
eCRF: electronic case report form
EOA: prosthesis opening area
IFU: Instructions for use
IQR: interquartile range
KCCQ: Kansas City Cardiomyopathy Questionnaire
LVEF: left ventricular ejection fraction
NYHA: New York Heart Association
PPM: patient prosthesis mismatch
PVL: paravalvular leakage
SAVR: surgical aortic valve replacement
SD: standard deviation
SF-12: Short Form-12 Health Survey
SVD: Structural valve degeneration
ViV: valve-in-valve
VTI: velocity time integral
